# Fixed-bearing is superior to mobile-bearing in lateral unicompartmental knee replacement: a retrospective matched-pairs analysis

**DOI:** 10.1007/s00167-023-07417-9

**Published:** 2023-04-24

**Authors:** Mustafa Hariri, Niklas Zahn, Paul Mick, Ayham Jaber, Tobias Reiner, Tobias Renkawitz, Moritz Innmann, Tilman Walker

**Affiliations:** Department of Orthopaedics, University Hospital of Heidelberg, Schlierbacher Landstrasse 200a, 69118 Heidelberg, Germany

**Keywords:** Unicompartmental knee replacement, Lateral, Knee arthroplasty, Survivorship, Osteoarthritis, Outcome

## Abstract

**Purpose:**

Due to low incidence of isolated lateral osteoarthritis (OA), there are limited data on whether a fixed-bearing (FB) or a mobile-bearing (MB) design is superior for lateral unicompartmental knee replacement (UKR). The aim of this matched-pairs analysis was to compare both designs in terms of implant survival and clinical outcome.

**Methods:**

Patients who received MB-UKR (Group A) and FB-UKR (Group B) at a single centre were matched according to gender, age at time of surgery and body mass index (BMI). Survivorship analysis was performed with the endpoint set as “revision for any reason”. Clinical outcome was assessed using the Oxford knee score (OKS), visual analogue scale for pain (VAS), patients’ satisfaction, University of California Los Angeles activity scale (UCLA) and the Tegner activity score (TAS).

**Results:**

A total of 60 matched pairs were included with a mean follow-up (FU) of 3.4 ± 1.3 (range 1.2–5.0) years in Group A and 2.7 ± 1.2 (range 1.0–5.0) years in Group B. Survivorship between both groups differed significantly (Group A: 78.7%; Group B: 98.3%, *p* = 0.003) with bearing dislocation being the most common reason for revision in Group A (46.2%). The relative and absolute risk reduction were 92.2% and 20%, respectively, with 5 being the number needed to treat. There were no differences in OKS (Group A: 41.6 ± 6.5; Group B: 40.4 ± 7.7), VAS (Group A: 2.9 ± 3.2; Group B: 1.6 ± 2.2), UCLA (Group A: 5.7 ± 1.3; Group B: 5.9 ± 1.8) and TAS (Group A: 3.0 ± 1.0; Group B: 3.1 ± 1.2) between both groups on follow-up.

**Conclusion:**

Despite modern prosthesis design and surgical technique, implant survival of lateral MB-UKR is lower than that of FB-UKR on the short- to mid-term due to bearing dislocation as the most common cause of failure. Since clinical results are equivalent in both groups, FB-UKR should be preferred in treatment of isolated lateral OA.

**Level of evidence:**

Retrospective case–control study, Level III.

## Introduction

After failure of joint-preserving methods, unicompartmental knee replacement (UKR) is recognised as a safe treatment option in end-stage unicompartimental knee osteoarthritis (OA) [29]. The indications for UKR have been extended to younger patients since initially introduced by Kozinn and Scott in 1989 [19, 30]. Due to several advantages compared to total knee replacement (TKR), such as a greater range-of-motion (ROM), faster recovery and lower perioperative morbidity, usage of UKR has increased over the last decades and is expected to increase even more [16, 20]. While many studies reported high survivorship and good clinical outcomes for mobile-bearing (MB) UKR in the medial compartment [8, 25, 27], initial results for lateral MB-UKR were disappointing with a survival rate of only 82% after 5 years [12]. The main cause of failure was dislocation of the bearing [12]. Further studies have attributed this contrast to the different anatomy and biomechanics of the lateral compared to the medial compartment [26, 37]. Therefore, the Oxford domed lateral (ODL) was introduced with a modified surgical technique to account for the aforementioned differences [28, 43]. However, bearing dislocation remains a recurrent complication with dislocation rates up to 8.5% [17, 34, 41]. As an alternative to the ODL prosthesis, the Oxford fixed lateral (OFL) prosthesis was developed as a fixed-bearing (FB) design, which also allows the use of a minimally invasive surgical technique [40]. To date, there are only few studies reporting the use of lateral FB-UKR with small cohorts and wide variations in clinical outcome and survivorship [22, 31, 33, 35]. Recent systematic analyses showed lowest revision rates for metal-backed FB-UKR in lateral UKR [9, 36]. However, these studies report on a variety of different prosthesis designs as well as register-based studies, that are known to include results from low-volume surgeons. Hence, there is a high risk that these results may be confounded by prosthesis choice as well as surgical technique, and experience. Additionally, there are currently no studies comparing clinical outcome of both designs in contemporary lateral UKR. To provide more evidence on the influence of bearing choice in lateral UKR, the aim of this study was to compare survivorship and clinical outcome in matched patients who underwent lateral UKR with either MB- or FB-UKR for isolated lateral OA at one institution using the same prosthesis and surgical technique.

The authors hypothesised that FB-UKR for isolated lateral OA would be superior to MB-UKR in terms of survivorship while demonstrating similar clinical outcome.

## Materials and methods

Ethical approval was obtained from the institutional review boards of the University of Heidelberg (S-265-2014, S-293-2021) and the study was conducted in accordance with the Helsinki Declaration of 1975, as revised in 2013. Informed consent was obtained from all participating patients.

The present study retrospectively analyses prospectively collected data from a series of patients who underwent UKR for isolated lateral OA in one institution. A total 258 UKR were implanted, subdivided into 115 MB-UKR (Group A) between 2006 and 2014 and 143 FB-UKR (Group B) between 2014 and 2020. In group A, the ODL (Zimmer Biomet Inc., Warsaw, Indiana, USA) was used as the MB-UKR and in group B, the OFL (Zimmer Biomet Inc., Warsaw, Indiana, USA) was used as the FB-UKR. Patients were assigned based on the timing of surgery, as the use of the ODL was discontinued in favour of the OFL in 2014 due to recurrent bearing dislocations at the authors institution.

To improve comparability, patients in groups A and B were matched according to gender, age at time of surgery and body mass index (BMI) in a 1:1 ratio. Patients were divided into six age groups (< 50, 50–54, 55–59, 60–64, 65–70, > 70 years) and three BMI groups (< 25, 25–30, > 30 kg/m^2^). To form matched pairs, patients had to be the same gender, in the same age and BMI group [39]. If more than two patients from both groups matched all three parameters, the pair with the closest age at the time of surgery was selected [1]. Matching was performed blinded with respect to outcome parameters and independently by two different authors (MH, TW), resulting in the same matched pairs.

To minimise a possible influence of large differences in follow-up (FU) duration between matches, only patients with a FU of at least 1 year and at most 5 years were included. Patients with missing postoperative data were excluded from the study.

In both groups, the primary indication for surgery was severe osteoarthritis of the lateral compartment with full thickness articular cartilage loss (“bone-on-bone”) or avascular necrosis of the femoral condyle. In all cases, the anterior cruciate ligament (ACL) as well as the medial (MCL) and lateral collateral ligaments (LCL) were functionally intact, the valgus deformity was manually correctable to ensure that no ligaments were rigid and there was no evidence of OA in the medial compartment on varus stress radiographs. OA of the patellofemoral joint was not considered a contraindication unless there was a deep eburnation or bone grooving on the medial facet of the patella. Rheumatoid arthritis, fixed valgus deformity, previous osteotomy, or a flexion deformity > 15° were considered contraindications [40].

All surgeries were performed using a minimally invasive surgical technique (MIS) through a lateral parapatellar approach without dislocation of the patella. Internal rotation of the tibial plateau and anatomical positioning of the femoral component were considered to avoid elevation of the joint line. Bearing thickness was selected in full extension. Depending on the bone quality, the use of a cemented or uncemented fixation of the femoral component was chosen, whereas the tibial component was always cemented in both groups [40, 41]. An intravenous single-shot antibiotic (1.5 g cefuroxime) was administered perioperatively. Postoperative rehabilitation was standardised for all patients. From the first postoperative day, immediate full weight bearing was possible. No restriction in active and passive knee movement was set. Discharge was followed by 3 weeks of inpatient or outpatient rehabilitation.

All procedures were performed by or under supervision of 8 senior surgeons with high experiences in unicompartmental knee replacement (≥ 15 UKR/year).

Survivorship analysis was performed with the endpoint “revision for any reason” defined as any operation in which at least one of the components was replaced.

The Oxford knee score (OKS) was obtained at the regular follow-up examination. These regular FU examinations are routinely performed at 1, 3 and 5 years postoperatively in all patients receiving an arthroplasty at our institution. Pain level was assessed using a visual analogue scale (VAS) ranging from 0 to 10 (0 = no pain, 10 = worst pain experienced). Postoperative satisfaction was evaluated using a numeric scale ranging from 1 (highly satisfied) to 5 (unsatisfied). The University of California Los Angeles activity scale (UCLA) and the Tegner activity score (TAS) were used to assess patients’ physical activity after surgery [5, 44]. Patients who were unable to attend the clinical FU were contacted by telephone for a structured interview to assess the aforementioned questionaries.

### Statistical analysis

Data were collected and analysed using SPSS version 29.0 (SPSS Inc., Chicago, IL). The primary endpoint was implant survivorship and secondary endpoints were clinical outcomes.

The empirical distribution of continuous variables was described using mean and standard deviation (SD), possible differences between the two groups were examined with the Mann–Whitney-*U* Test and differences between preoperative and postoperative values were analysed with the Wilcoxon signed rank test. Survivorship analysis was performed with the Kaplan–Meier estimator. Survival rates between the two groups were compared using the log-rank test. For all tests, the significance level was set at *p* < 0.05. A priori power analysis for medium effect size with a type 1 error (two sided) of 0.05 and a power of 80% yielded a minimum number of 45 cases for each group.

## Results

**Fig. 1:**
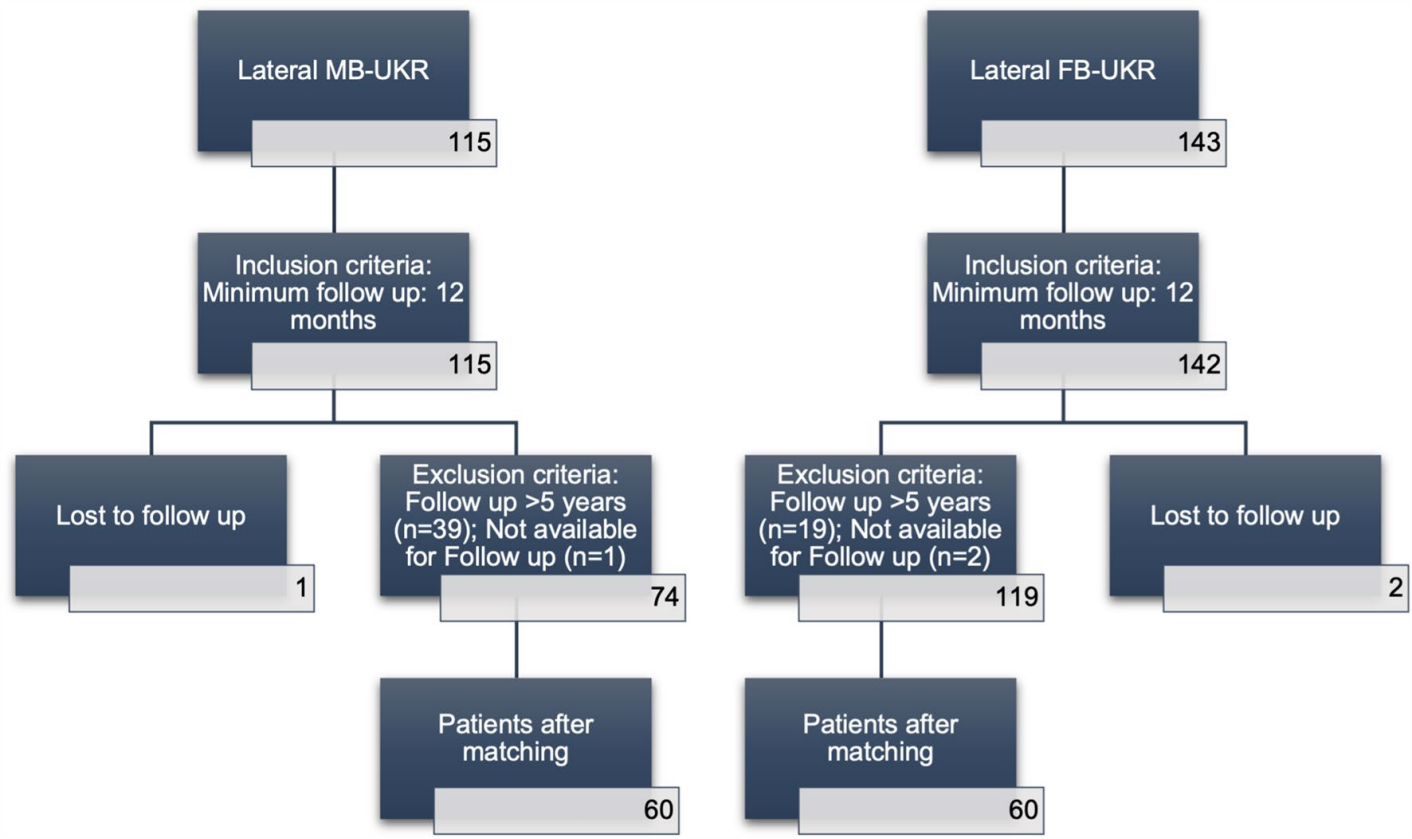
Flow chart illustrating number of patients who met the inclusion and exclusion criteria. After matching by age at time of surgery, body mass index and gender, 60 matched pairs were build. *MB* mobile bearing, *FB* fixed bearing, *UKR* unicompartmental knee replacement

A total of 60 matched pairs were included in the analysis after matching with gender, age at time of surgery and BMI (Fig. 1). These matching parameters showed no significant differences between both groups. In addition, the mean preoperative OKS between both groups showed no significant difference (*p* < 0.05, Table 1). Patient demographics are shown in Table 1.

**Table 1:** Patient demographics for each group

Demographics	Group A (ODL)	Group B (OFL)	*p* value
Number of patients	60	60	–
Gender (%)	Female 49 (81.7%); Male 11 (18.3%)	Female 49 (81.7%); Male 11 (18.3%)	–
Mean age at time of surgery in years (± SD)	61.3 ± 10.3	61.4 ± 11.2	0.933
Mean body mass index (kg/m^2^) (± SD)	27.8 ± 5.6	27.5 ± 6.0	0.725
Mean follow-up in years (± SD)	3.4 ± 1.3	2.7 ± 1.2	0.004
Preoperative OKS (± SD)	28.1 ± 8.2	27.3 ± 7.8	0.600

*ODL* Oxford domed lateral, *OFL* Oxford fixed lateral, *OKS* Oxford knee score, *SD* standard deviation

### Survivorship analysis

**Fig. 2:**
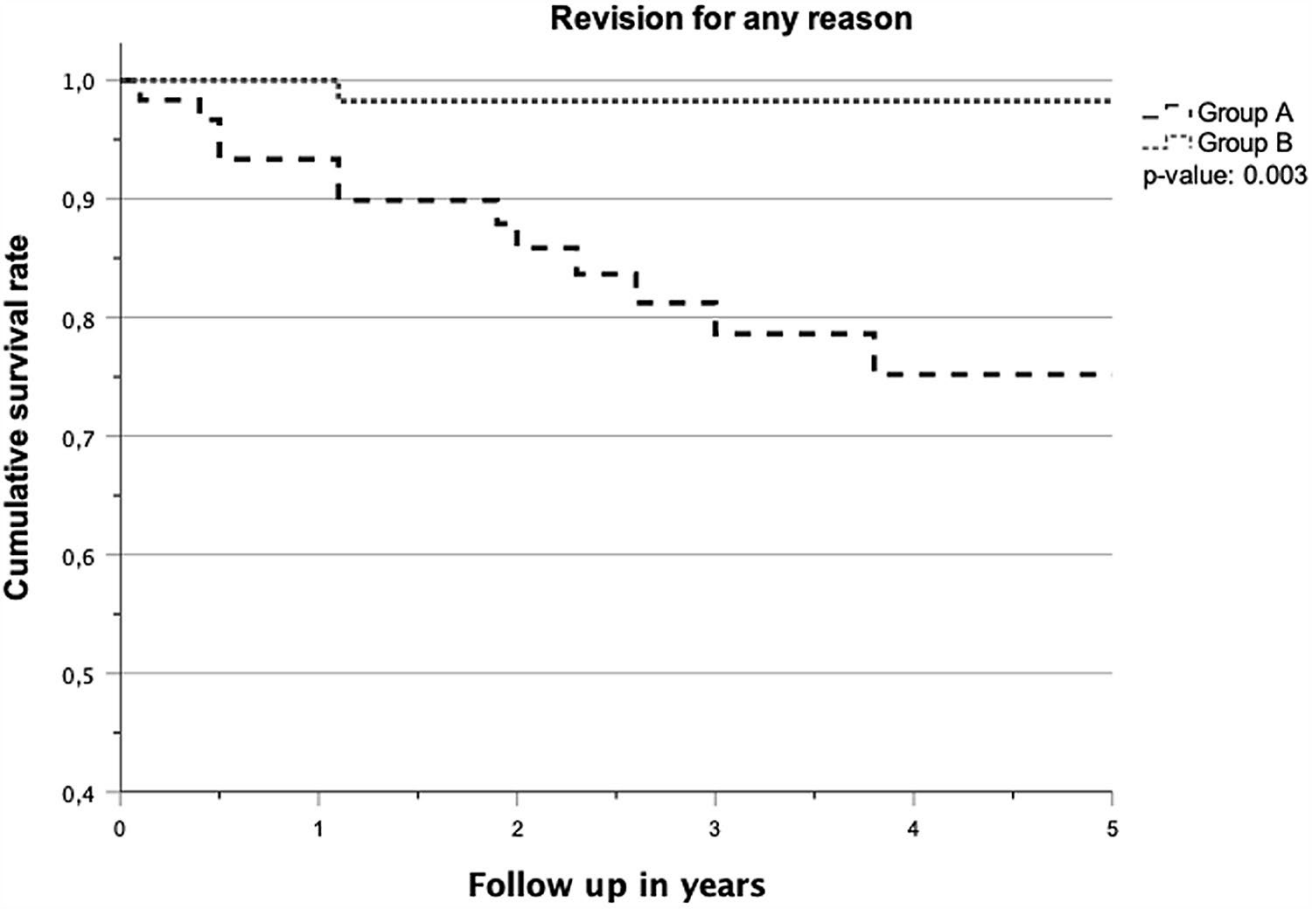
Kaplan–Meier survivorship curve for “revision for any reason” as the endpoint for both groups. A significant difference was observed in the log-rank test (*p* = 0.003)

There were 14 revision surgeries in the present study, of which 13 (21.7%) were in group A and one (1.7%) in group B. Considering these event rates in both groups, we calculated a relative risk reduction of 92.2% and an absolute risk reduction of 20.0% resulting in a number needed to treat of 5, which means that 5 patients have to be treated with lateral FB-UKR to prevent one patient from having revision surgery. This results in a survival rate of 78.7% at 3.4 years (number at risk: 28) for group A and a survival rate of 98.3% at 2.7 years (number at risk: 26) for group B. Survival rates between both groups showed a significant difference (*p* = 0.003) (Fig. 2).

The different reasons for revision are demonstrated in Table 2.

**Table 2:** Reasons for revision in group A and group B

Reason for revision	Group A (ODL) (*n*/%)	Group B (OFL) (*n*/%)
Aseptic loosening	1 (7.7%)	–
Infection	2 (15.4%)	–
Progressive OA	1 (7.7%)	–
Persistence of pain	3 (23.1%)	1 (100%)
Bearing dislocation	6 (46.2%)	–
Total	13 (100%)	1 (100%)

*OA* Osteoarthritis, *ODL* Oxford domed lateral, *OFL* Oxford fixed lateral

Treatment options in Group A included revision to TKR (7 cases/53.8%), bearing exchange (3 cases/23.1%), revision to the FB design (2 cases/ 15.4%) and screw insertion into the intercondylar notch (1 case/ 7.7%). In group B, revision to TKR was performed.

### Clinical outcome

**Fig. 3:**
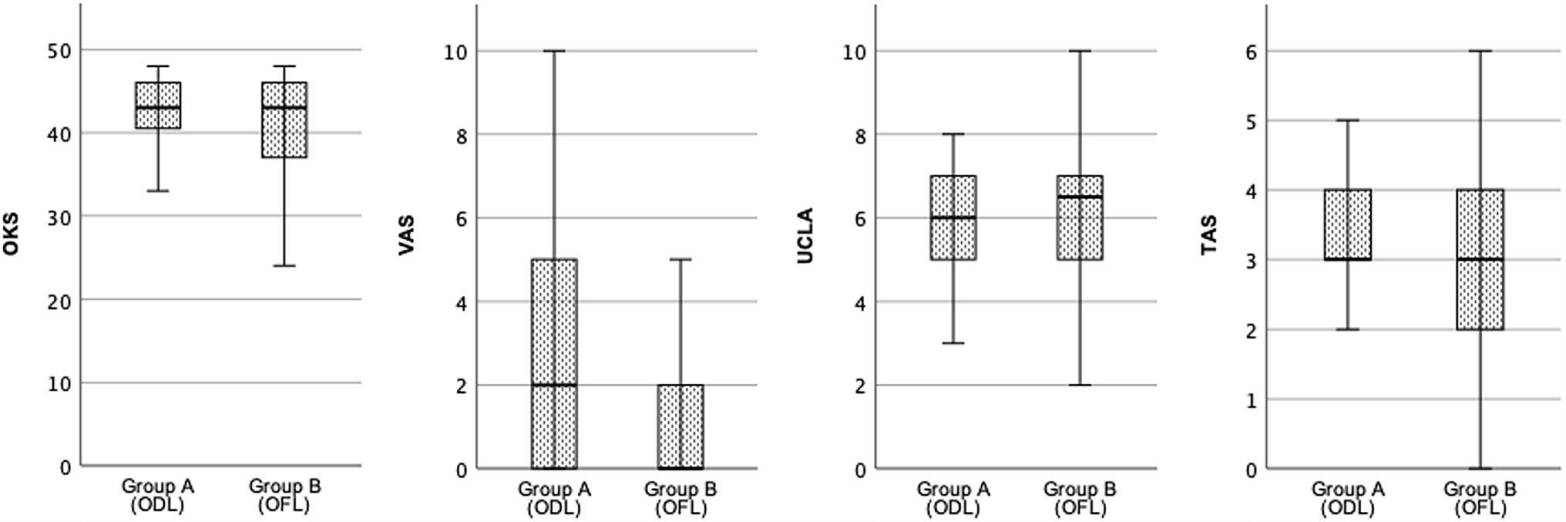
Clinical outcome at last follow-up showing no differences between both groups. *OKS* Oxford knee score, *VAS* Visual analogue scale, *UCLA* University of California Los Angeles activity scale, *TAS* Tegner activity score, *ODL* Oxford domed lateral, *OFL* Oxford fixed lateral

While there was a significant improvement in the OKS from pre- to postoperative values in both groups, there were no significant differences in the postoperative patient-reported outcome measurements (PROMs) between the two groups at final FU (Table 3, Fig. 3).

**Table 3:** Postoperative PROMs for both groups. There were no significant differences between both groups

	Group A (ODL)	Group B (OFL)	*p* value
OKS (Mean ± SD)	41.6 ± 6.5	40.4 ± 7.7	0.544
VAS (Mean ± SD)	2.9 ± 3.2	1.6 ± 2.2	0.059
UCLA (Mean ± SD)	5.7 ± 1.3	5.9 ± 1.8	0.333
TAS (Mean ± SD)	3.0 ± 1.0	3.1 ± 1.2	0.929

*OKS* Oxford knee score, *VAS* visual analogue scale, *UCLA* The University of California Los Angeles activity scale, *TAS* Tegner activity score, *ODL* Oxford domed lateral, *OFL* Oxford fixed lateral, *SD* standard deviation

**Fig. 4:**
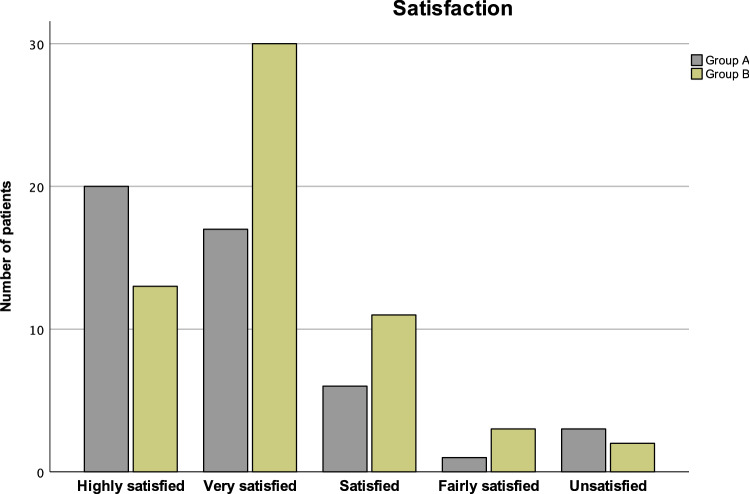
Patients’ satisfaction in both groups at last follow-up

There was no significant difference in patients’ satisfaction between the two groups (*p* = 0.078, Fig. 4).

## Discussion

The main finding of the present study is that implant survival with the endpoint “revision for any reason” following lateral FB-UKR is significantly better than following lateral MB-UKR while demonstrating similar clinical results.

This is the first comparative study to analyse the influence of bearing choice in lateral UKR on survivorship and clinical outcome in a single high-volume institution using the same prosthesis.

The findings of this study are consistent with the aforementioned systematic analyses, in which metal-backed FB-UKR showed lowest revision rates for lateral UKR [9, 36].

Almost half of the revisions in group A were due to bearing dislocation despite the use of the modified surgical technique described earlier [28, 32]. The dislocation rate of 10% is noticeable higher than in previous reports, even if all surgeries were performed by well-experienced senior surgeons who were familiar with the modified surgical technique [41, 42]. In contrast, survivorship of FB-UKR for the endpoint “revision for any reason” was significantly higher with 98.3% at 2.7 years than for MB-UKR and is in line with other recent studies of lateral FB-UKR [3, 35].

A common reason for revision in both groups was persistence of pain. Objective causes of revision such as aseptic loosening, infection, instability, or OA progression could not be identified in any of the present cases. Treatment of these patients remains challenging since the threshold for revision surgery in UKR is lower than in TKR, even in patients with similar OKS scores [21] and the outcome after revision surgery for unexplained pain is worse than revision for an identified reason [18].

OA progression and aseptic loosening are usually causes of failure in late years, so the FU period in the present study may not be representative. Still, there was one case of each in group A in early years. Overcorrection of valgus malalignment is associated with a higher risk of OA progression and may be considered as a possible explanation in this case [2]. Burger et al. demonstrated higher revision rates due to OA progression in lateral MB-UKR than in lateral FB-UKR and concluded that there is a tendency to overstuff the lateral compartment to prevent bearing dislocation [7]. In contrast, overstuffing may also lead to knee laxity due to soft tissue stretching, which increases the risk of bearing dislocation [11]. This highlights the importance of precise component alignment and ligamentous balancing in MB-UKR, suggesting that this design is more prone to surgeon related errors [14]. Since bearing dislocation is not possible with a FB design, the requirements for ligamentous balancing are not as crucial as for MB-UKR. In recent years, robotic-assisted UKR has emerged and has shown improved implant positioning compared with the conventional technique [4] as well as good clinical outcome and survivorship [10, 15]. However, an advantage of robotic-assisted lateral UKR over conventional technique in terms of survivorship and clinical outcome has not yet been demonstrated [23]. To what extent robotics can optimise intraoperative balancing to decrease bearing dislocations remains unclear and needs to be further investigated.

There were no significant differences between the two groups in the clinical results. Both achieved high mean OKS scores postoperatively consistent with previous results for lateral MB-UKR [28, 34, 42], lateral FB-UKR [3, 35] as well as medial UKR [24]. Furthermore, no significant differences were found in postoperative pain level and patients’ satisfaction between both groups demonstrating equivalent clinical results for both designs despite the theoretical biomechanical advantages of MB-UKR. Similar results were demonstrated by Burger et al. in a systematic analysis of 28 studies involving 2265 lateral UKRs [6].

One aim of UKR is to restore patients’ activity level, which is not always consistent with clinical outcomes [44]. The current literature provides limited data on patient’s activity level after lateral UKR, but shows that moderate activity levels are possible, especially in low impact activities such as cycling, swimming, and hiking [13, 38, 45]. This study shows no differences in the activity rating scales assessed between both groups and, therefore, suggests that both designs enable good activity levels after UKR.

This study has several limitations. First, a small sample size was reported in a retrospective study design with a well-known risk of selection bias. Randomised controlled studies are generally preferable, but because of the rare surgical indications of isolated lateral OA, they are unlikely to be practically feasible with good power.

Second, the FU period is limited to a short- to mid-term period, so causes of failure in late years, such as OA progression and aseptic loosening, may be not representative in this study and a longer FU is necessary. Third, PROMs were collected at different time points, ranging from 1 to 5 years. Collecting data at the same timepoint would have strengthened the data.

Fourth, radiographic analysis of alignment and implant positioning was not reported because it was not the purpose this study. Nevertheless, this could lead to a better understanding in patients who need revision surgery or whose clinical outcome is worse and should be further investigated in the future. Fifth, the number of patients who received different treatment because of concomitant patellofemoral OA during the study period is unknown. Since the extent of patellofemoral OA is not always clear, the decision may have varied from surgeon to surgeon in some cases. However, it is not expected to have had a significant influence on the results of this study. Finally, this multi-surgeon study comprises results from a single centre with high experience in UKR. Multicenter or registry-based studies would constitute more generalised data. Nevertheless, lateral UKR may never be a widely used treatment option since its indication is very rare and surgical procedure is different than for medial UKR.

## Conclusion

Despite modern prosthesis design and surgical technique, implant survival of lateral MB-UKR is lower than that of FB-UKR on the short- to mid-term due to bearing dislocation as the most common cause of failure. Since clinical results are equivalent in both groups, FB-UKR should be preferred in treatment of isolated lateral OA.

## Data Availability

The datasets used and analyzed during the current study are available from the corresponding author on reasonable request.

## Abbreviations

ACL: Anterior cruciate ligament
FB: Fixed bearing
FU: Follow-up
LCL: Lateral collateral ligament
MB: Mobile bearing
MCL: Medial collateral ligament
MIS: Minimally invasive surgical technique
OA: Osteoarthritis
ODL: Oxford domed lateral
OFL: Oxford fixed lateral
OKS: Oxford knee score
PROM: Patient-reported outcome measurements
ROM: Range-of-motion
TAS: Tegner activity score
TKR: Total knee replacement
UCLA: The University of California Los Angeles activity scale
UKR: Unicompartmental knee replacement
VAS: Visual analogue scale for pain
